# Supplementary material for: Cholinergic interneuron control of GABAergic circuits targeting spiny projection neurons is disrupted in parkinsonian models
Source: bioRxiv. 2026 Jun 22:2026.06.17.732978. Preprint. [Version 1] doi: 10.64898/2026.06.17.732978 (PMC13320840; doi:10.64898/2026.06.17.732978)

Figure S1. (a) Morphology of a reconstructed dSPN. (b) Plot of input resistance (measured using hyperpolarizing current steps of −10 pA, 1000 ms) as a function of holding membrane potential under control conditions (black, filled circles) and following partial block of K_v7 potassium channels (80% reduction in local membrane conductance density where expressed; red, open circles). (c) Plot of membrane conductance density versus holding membrane potential. At hyperpolarized potentials, input resistance is dominated by high $K_{ir}2$ conductance density. The increase in input resistance with depolarization reflects the intrinsic inward rectification of this channel, manifested as a reduction in $K_{ir}2$ conductance density. At more depolarized potentials, the subsequent decrease in input resistance is primarily attributable to activation of other voltage-gated K^+ channels, notably K_v4 and Kv1 increasing their conductance densities. K_v7 blockade only shifts its conductance density (open circles; direction indicated by an arrow). (d) Rheobase (defined as the minimum positive current to cause an action potential from membrane resting potential) was +155 pA under control (black line). The same current step causes a dramatic increase in spiking to the same current step following partial block of K_v7 potassium channel (80% reduction; red line). (e) Single examples of increasing positive current steps and (f) summary to show the reduced rheobase and increased spike count rate to depolarizing current steps when K_v7 is blocked (control: black, filled circles; K_v7 blockade: red, open circles). Similar plots are shown for the simulated iSPN (g-l). The effects are qualitatively similar. The main differences are that input resistance is slightly elevated manifesting as an increased excitability / reduced rheobase (iSPN vs dSPN: +129 pA vs +155 pA).

Figure S2. Effect of M_1 mAChR activation on the temporal interaction between subthreshold glutamatergic and GABAergic inputs in a model iSPN. (a) Reconstructed iSPN morphology showing clustered glutamatergic input (red) and fast synaptic GABAergic input (blue). (b) Effect of increasing glutamatergic synapse number at a fixed dendritic site with fast synaptic GABAergic input (12 synapses each comprising 40 receptors with 25 pS peak conductance; Σg_{GABA} = 12 nS; PSP amplitude 8.0 mV at dendritic spike site) in control (left) and M_1 receptor modulation of $K_{ir}2$, K_v4 and K_v7 K^+ channels (right). Color traces represent incremental

bioRxiv preprint doi: https://doi.org/10.64898/2026.06.17.732978; this version posted June 22, 2026. The copyright holder for this preprint (which was not certified by peer review) is the author/funder, who has granted bioRxiv a license to display the preprint in perpetuity. It is made available under a CC-BY 4.0 International license.

glutamatergic recruitment (N_{GLUT} = 0-16) with fixed GABAergic activity (Δt = +10 ms; *i.e.* glutamate activity follows GABAergic input) at dendritic site of spike generation and cell soma. **M_1** receptor activation lowers dendritic spike threshold from N_{GLUT} = 11 to 9. (c) Reconstructed iSPN morphology showing dendritic sites of clustered glutamatergic activation (red) and slow (green) extrasynaptic GABAergic receptor inputs. (d) Illustrating the effect of the slow GABAergic activation (170 receptors, 25 pS; Σg_{GABA} = 4.25 nS; PSP = 7.3 mV at dendritic spike site) on the same dendritic glutamatergic inputs as before (i.e. panel B; Δt = +30 ms). Again, M_1 receptor activation lowers dendritic spike threshold from N_{GLUT} = 11 to 9.

Figure S3. Effect of M_1 mAChR activation on the interaction between suprathreshold glutamatergic input and varying amplitude, temporally fixed GABAergic input in a model iSPN. (a) Reconstructed iSPN morphology showing four dendritic sites of clustered glutamatergic activation (red; sites 1-4) and fast synaptic GABAergic input (blue). (b) Synaptic potentials at a dendritic site (site 1) and soma showing the effect of varying fast synaptic GABAergic input alone (gray) and when delivered 30 ms after suprathreshold glutamatergic input (14 inputs/site; color traces). Traces illustrate the effect of increasing GABAergic synaptic activity (0 - 48 synapses; each comprising 40 receptors with 25 pS peak conductance; blue to red) delivered at the same temporal offset (*i.e.* Δt = -30 ms) in the absence and presence of M_1R activation. GABAergic synapse recruitment increased somatic depolarization (blue open circles; dotted line) with a concomitant decrease in impedance (orange open circles; dotted line). A small reduction in the total number of spikes generated at the soma was observed (blue open circles; dotted line). M_1 receptor modulation of $K_{ir}2$, K_v4 and K_v7 K^+ channels increased GABAergic-mediated somatic depolarization and maintained somatic spike output (closed circles; solid line). (c, d) As in (a, b) but for slow extrasynaptic GABAergic receptor activation (0 - 1920 receptors; blue to red) color-matched to the same overall conductance loads as panel c (*i.e.* Σg_{GABA}) at the same temporal offset *(i.e.* Δt = -30 ms). Extrasynaptic GABAergic receptor recruitment increased somatic depolarization (blue open circles; dotted line) with a concomitant decrease in impedance (orange open circles; dotted line). The suppression of spiking and its relief by M_1R activation were more pronounced with slow GABAergic activation than fast synaptic activation at the same overall conductance load.

bioRxiv preprint doi: https://doi.org/10.64898/2026.06.17.732978; this version posted June 22, 2026. The copyright holder for this preprint (which was not certified by peer review) is the author/funder, who has granted bioRxiv a license to display the preprint in perpetuity. It is made available under a CC-BY 4.0 International license.

Figure S4. Effect of M_1 mAChR activation on the interaction between glutamatergic input and fast synaptic GABAergic responses in a model dSPN. (a) Circuit diagram depicting cholinergic control of GABAergic microcircuits targeting SPNs and a schematic illustrating inputs onto a stretch of SPN dendrite. (b) Reconstructed dSPN morphology showing subthreshold (1 site) and suprathreshold (4 sites) clustered glutamatergic activation (red) and fast synaptic GABAergic input (blue). **(c)** Effect of fast synaptic GABAergic activation on glutamatergic synaptic potentials at dendritic and somatic sites in control (left) and following M_1 receptor modulation of K_v7 channels (right). Black trace: subthreshold glutamatergic excitation (12 inputs). Gray trace: fast GABAergic synaptic activation (12 synapses each comprising 40 receptors with 25 pS peak conductance; Σg_{GABA} = 12 nS; PSP = 6.3 mV at dendritic spike site). Colored traces (blue to red): glutamatergic-GABAergic interaction at temporal offsets $\Delta t = t_{GLUT} - t_{GABA}$ is illustrated from -30 to 150 ms; 10 ms intervals. (d) Summary of timing dependent effects on PSP amplitude. No effect of M_1 receptor activation was observed. (e, f) As in (c, d) but for suprathreshold glutamatergic input. Fast synaptic GABAergic input reduced somatic output with only modest effects of M_1 receptor mediated modulation. As in (c, d) but for suprathreshold glutamatergic input (15 inputs per site). Gray trace: fast synaptic GABAergic activation (40 synapses each comprising 40 receptors with 25 pS peak conductance; Σg_{GABA} = 40 nS). Colored traces (blue to red): glutamatergic-GABAergic interaction at temporal offsets $\Delta t = t_{GLUT} - t_{GABA}$ of -100, -80, -60, -40, -20, respectively. Only a modest effect of M_1 receptor activation on spike output was observed.

Figure S5. ChI-evoked GABAergic input in SPNs was reduced following 6-OHDA lesion in independent study. (a) Schematic of opsin injection into the DLS together with unilateral 6-OHDA injection into the substantia nigra pars compacta (SNc) of ChAT-ChR2-eYFP mice. Representative PSCs recorded from SPNs in control (b) and 6-OHDA (c) conditions in response to activation of ChIs (whole-field LED, 5 ms), showing fast- and slow-decaying gabazine-sensitive components. Insets, semi-logarithmic scale. (d) Semi-log box plots of PSC amplitude and charge transfer in SPNs (control: n = 21, 9 animals; 6-OHDA: n = 21, 9 animals) showing reduced responses after 6-OHDA (Wilcoxon rank-sum test, control vs 6-OHDA: amplitude, W = 39, $p = 6.3 \times 10^{-7}$; charge transfer, W = 404, $p = 4.3 \times 10^{-7}$). (e) Scatter plots of slow-decaying versus fast-decaying amplitudes in control (black) and 6-OHDA (red) conditions. (f) Semi-log box plots of fast and slow decay time constants in SPNs showing no change in kinetics

bioRxiv preprint doi: https://doi.org/10.64898/2026.06.17.732978; this version posted June 22, 2026. The copyright holder for this preprint (which was not certified by peer review) is the author/funder, who has granted bioRxiv a license to display the preprint in perpetuity. It is made available under a CC-BY 4.0 International license.

(Wilcoxon rank-sum test, control vs 6-OHDA: fast, W = 240, p = 0.31292; slow, W = 188, p = 0.76828). As before, im some recordings, only one component (fast or slow) was identified (see Methods for the statistical criterion). These ‘unpaired’ decays are shown in red without a corresponding value in the other condition. Displayed n values are adjusted accordingly.

Figure S6. ChI-evoked input to TH+ neurons was reduced following 6-OHDA lesion. (a) In situ hybridization showing CHRNB2 (β2 subunit) mRNA expression in TH^+ neurons under control and 6-OHDA conditions. (b) Box plots of β2-containing nicotinic acetylcholine receptor (nAChR) expression in TH^+ neurons, demonstrating reduced expression following 6-OHDA (robust linear mixed-effects model, Nboot = 1,999, $p < 0.0005$).

bioRxiv preprint doi: https://doi.org/10.64898/2026.06.17.732978; this version posted June 22, 2026. The copyright holder for this preprint (which was not certified by peer review) is the author/funder, who has granted bioRxiv a license to display the preprint in perpetuity. It is made available under a CC-BY 4.0 International license.

Figure S1

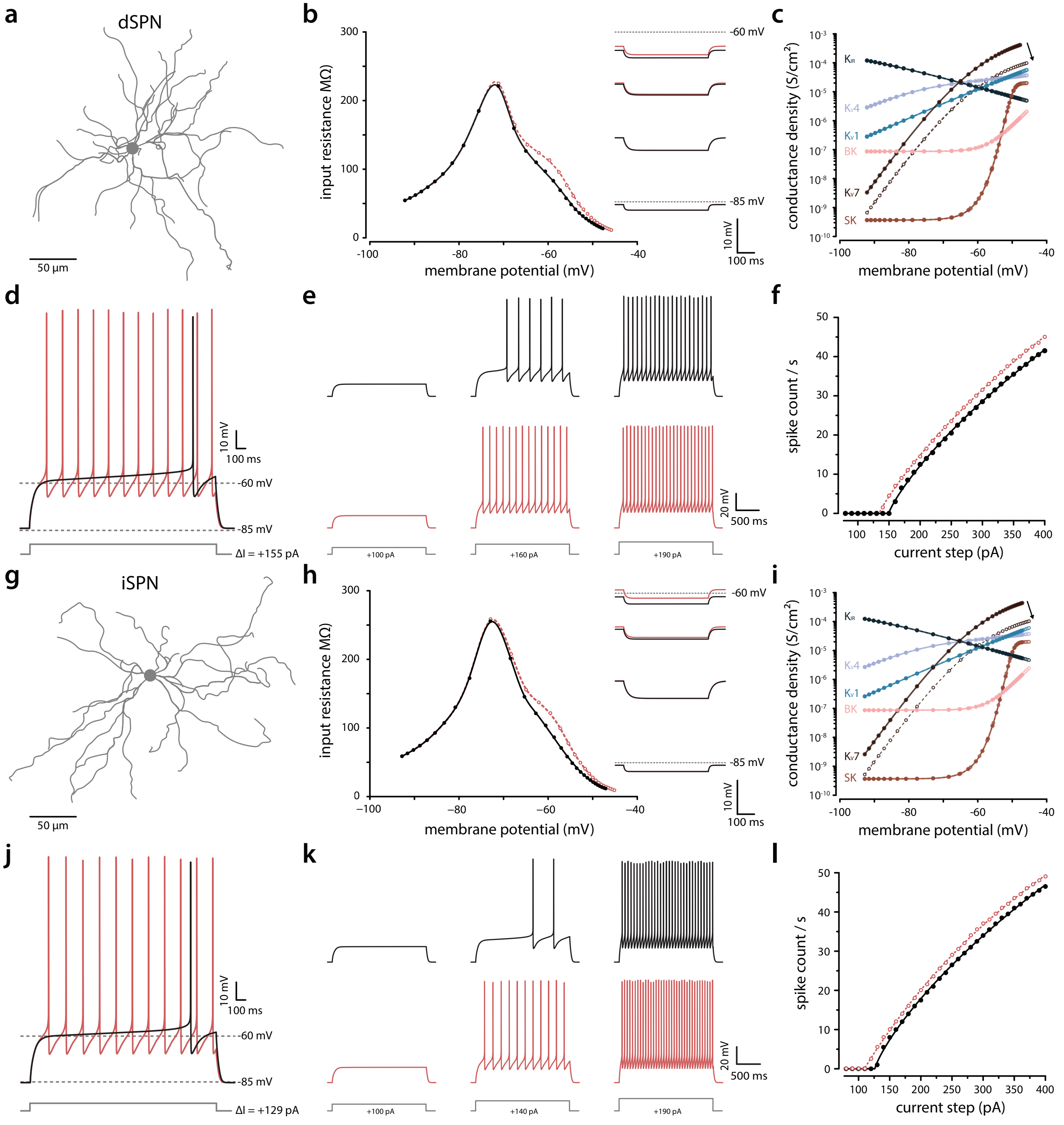

bioRxiv preprint doi: https://doi.org/10.64898/2026.06.17.732978; this version posted June 22, 2026. The copyright holder for this preprint (which was not certified by peer review) is the author/funder, who has granted bioRxiv a license to display the preprint in perpetuity. It is made available under a CC-BY 4.0 International license.

Figure S2

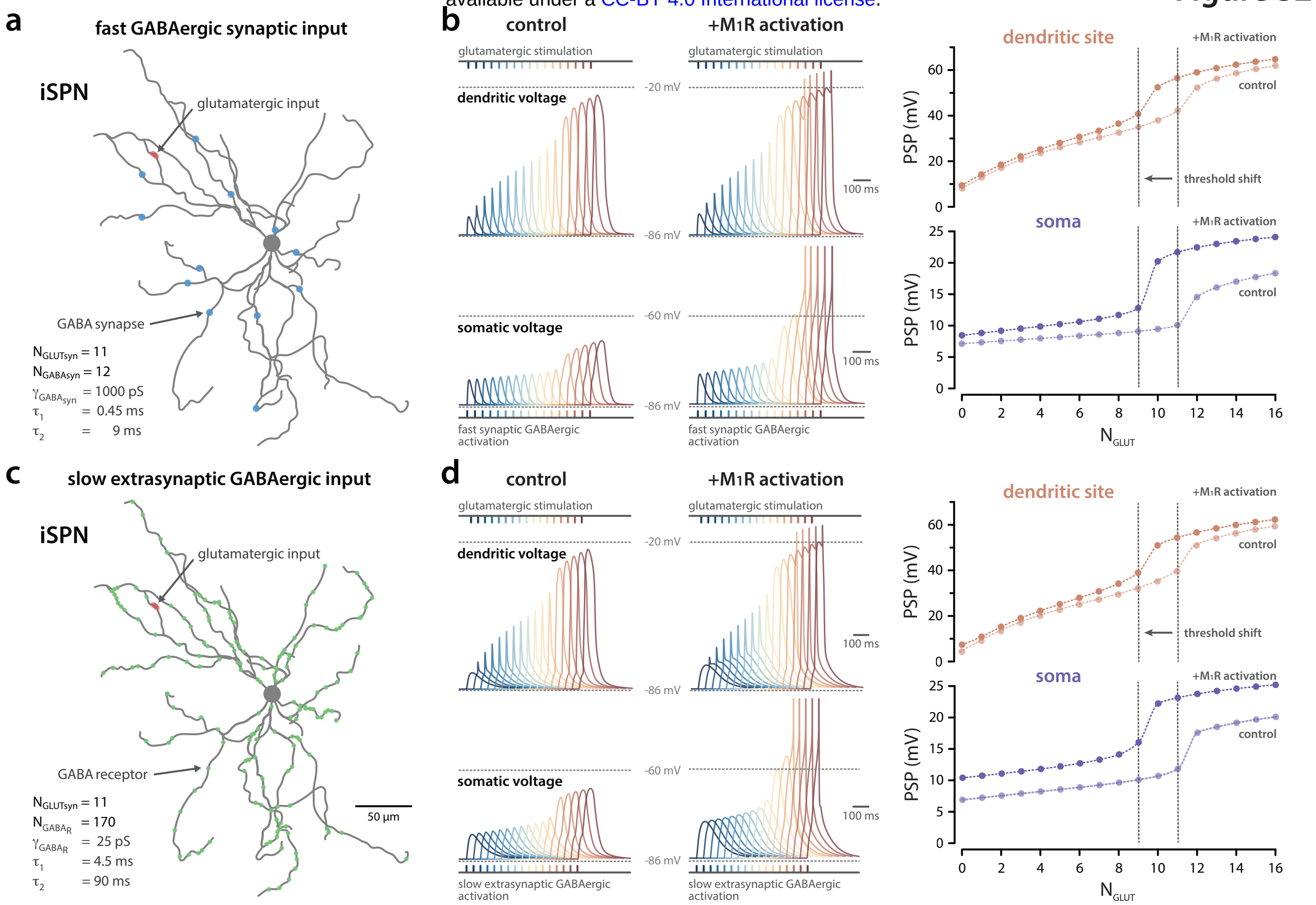

bioRxiv preprint doi: https://doi.org/10.64898/2026.06.17.732978; this version posted June 22, 2026. The copyright holder for this preprint (which was not certified by peer review) is the author/funder, who has granted bioRxiv a license to display the preprint in perpetuity. It is made available under a CC-BY 4.0 International license.

Figure S3

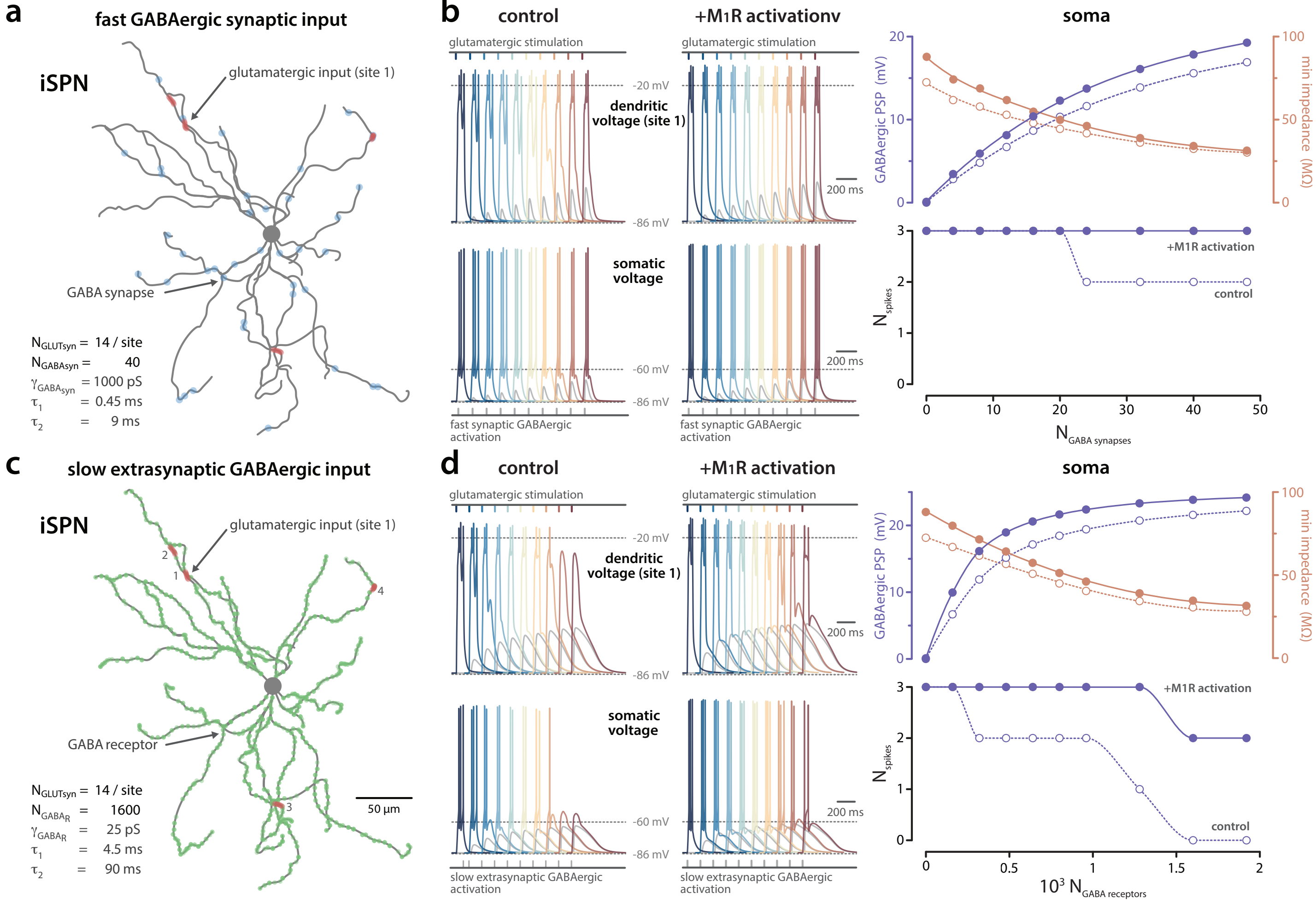

bioRxiv preprint doi: https://doi.org/10.64898/2026.06.17.732978; this version posted June 22, 2026. The copyright holder for this preprint (which was not certified by peer review) is the author/funder, who has granted bioRxiv a license to display the preprint in perpetuity. It is made available under a CC-BY 4.0 International license.

Figure S4

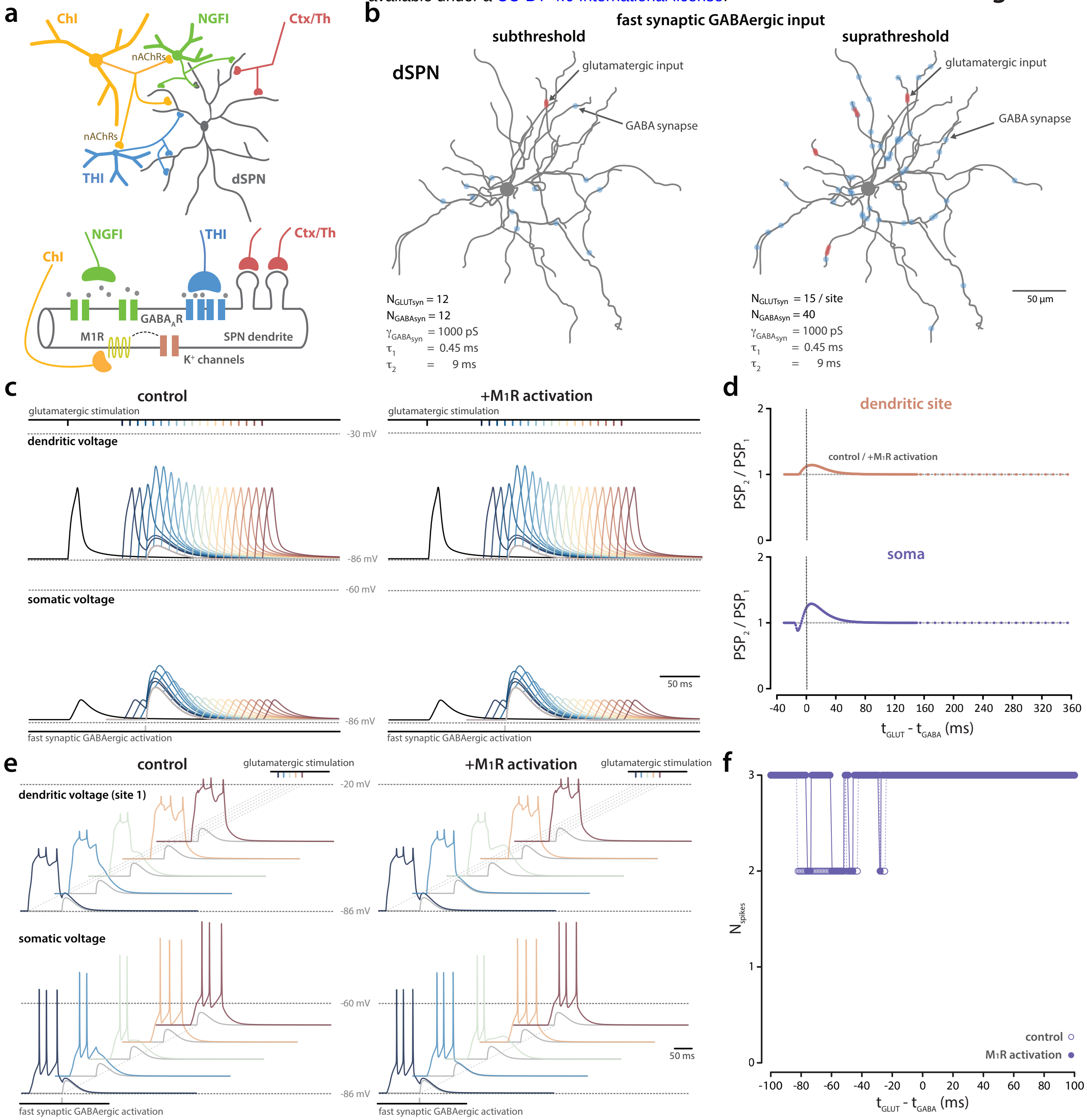

bioRxiv preprint doi: https://doi.org/10.64898/2026.06.17.732978; this version posted June 22, 2026. The copyright holder for this preprint (which was not certified by peer review) is the author/funder, who has granted bioRxiv a license to display the preprint in perpetuity. It is made available under a CC-BY 4.0 International license.

Figure S5

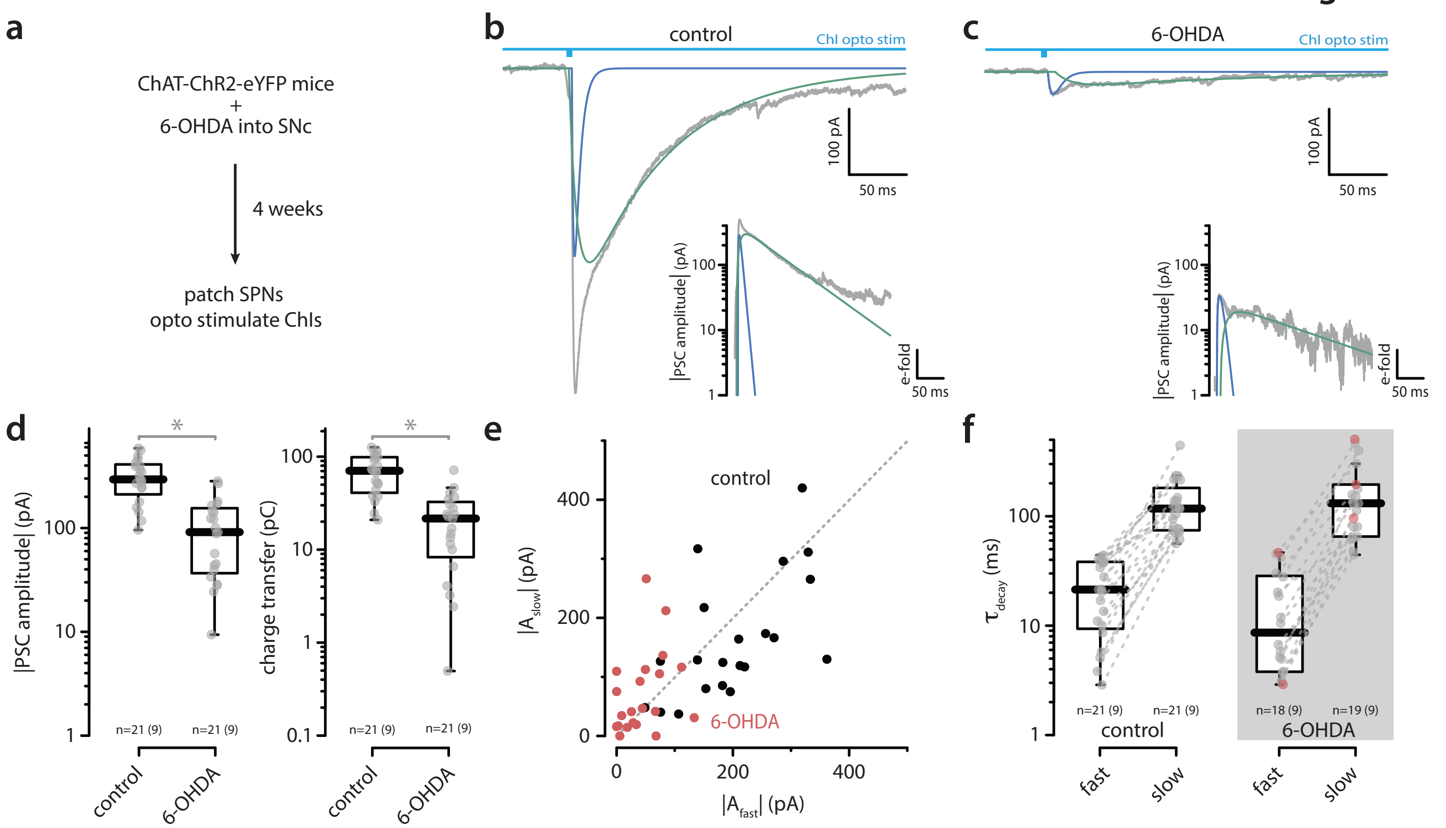

bioRxiv preprint doi: https://doi.org/10.64898/2026.06.17.732978; this version posted June 22, 2026. The copyright holder for this preprint (which was not certified by peer review) is the author/funder, who has granted bioRxiv a license to display the preprint in perpetuity. It is made available under a CC-BY 4.0 International license.

Figure S6

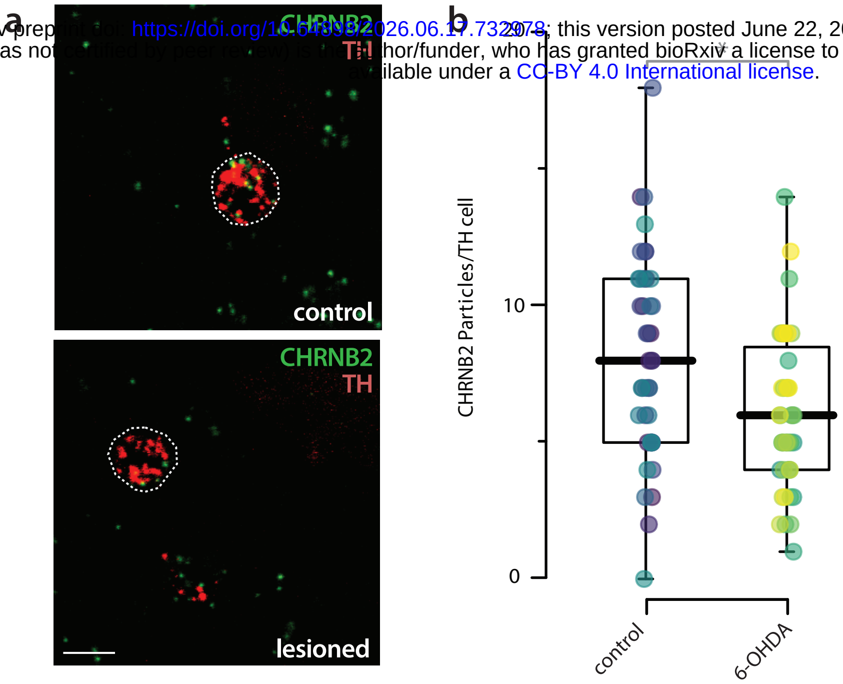

Supplement: Supplement 1 [file NIHPP2026.06.17.732978v1-supplement-1.pdf]
